# Patent Ductus Arteriosus Clinical Trials: Lessons Learned and Future Directions

**DOI:** 10.3390/children8010047

**Published:** 2021-01-15

**Authors:** Aisling Smith, Afif EL-Khuffash

**Affiliations:** 1 Department of Neonatology, The Rotunda Hospital, DO1 P5W9 Dublin, Ireland; smithai@tcd.ie; 2 Department of Paediatrics, The Royal College of Surgeons in Ireland, DO1 P5W9 Dublin, Ireland

**Keywords:** patent ductus arteriosus, premature infants, randomised controlled trials, outcomes

## Abstract

The identification of an optimal management strategy for the patent ductus arteriosus (PDA) in the context of extreme prematurity remains elusive. Observational studies have reported a persistent association between PDA and neonatal adverse outcomes, but by and large, no clinical trial, to date, has demonstrated that treating a PDA results in a reduction of those morbidities. This discrepancy has led many to assume that the PDA is an innocent bystander in the physiological mechanisms responsible for such complications and a reluctance to actively pursue shunt elimination. It would be remiss to discount the volume of evidence available clearly documenting a strong association between longstanding PDA exposure and negative outcomes. There needs to be a radical change in the design, patient selection and possible outcome assessment in any further trials addressing the PDA. The purpose of this review is to explore the reasons that preclude existing clinical trials from definitively ascribing a causal relationship between PDA patency and adverse outcomes in the context of extreme prematurity, why previous studies have failed to demonstrate significant beneficial effects following PDA treatment and how future research may be conducted to allow us to draw concrete conclusions regarding the potential merits of ductal closure.

## 1. Introduction

Despite decades of effort by neonatologists, the identification of an optimal management strategy for the patent ductus arteriosus (PDA) in the context of extreme prematurity remains elusive. At the heart of this ongoing controversy lies the inability to definitively establish a cause-and-effect relationship between PDA and significant adverse clinical outcomes [1]. Although many observational studies have reported a persistent association between PDA and neonatal morbidities, including chronic lung disease (CLD), necrotising enterocolitis (NEC), acute kidney injury (AKI) and intraventricular haemorrhage (IVH), no clinical trial, to date, has demonstrated that treating a PDA results in a reduction of those morbidities (with the possible exception of IVH) [2,3,4]. This discrepancy has led many to assume that the PDA is an innocent bystander in the physiological mechanisms responsible for such complications and a reluctance to actively pursue shunt elimination. Advocates of the conservative approach to PDA management reasonably argue that without verification of PDA pathogenicity, exposure to medical and surgical interventions aimed at ductal closure are unwarranted [5,6,7].

It would be remiss to discount the volume of evidence available clearly documenting a strong association between longstanding PDA exposure and negative outcomes. Further exploration of this association should be pursued. However, there needs to be a radical change in the design, patient selection and possible outcome assessment in any further trials addressing the PDA. The purpose of this review is to explore the reasons that preclude existing clinical trials from definitively ascribing a causal relationship between PDA patency and adverse outcomes in the context of extreme prematurity, why previous studies have failed to demonstrate significant beneficial effects following PDA treatment and how future research may be conducted to allow us to draw concrete conclusions regarding the potential merits of ductal closure. 

## 2. Challenges with PDA Trials to Date

At the core of this incongruity are several consistent problems with how previous randomised control trials (RCTs) and observational studies have been designed, performed, analysed and interpreted. Such issues mar our understanding of the natural course of the PDA, whether a causal relationship does exist between ductal patency and serious morbidity and if shunt elimination successfully ameliorates such negative consequences. Key matters include the absence of consensus on what constitutes ‘haemodynamic significance’ of the PDA, the heterogeneous response to medical therapy for the PDA among extremely premature babies, analysis of PDA RCTs on an intention to treat basis rather than an assessment of successful shunt elimination in the intervention arm and a predilection for over-emphasising the importance of long term outcomes over short term sequela [8,9].

### 2.1. Defining Haemodynamic Significance in PDA Trials

The cardiovascular consequences of a PDA are now well-documented. Following a fall in pulmonary vascular resistance (PVR) over the first 12 to 24 h after delivery, shunt volume and blood flow across the ductus from the systemic to the pulmonary circulation is increased [10]. The flow across the shunt is governed by Poiseuille’s Law, which states that: “At a constant driving pressure [the pressure gradients across the PDA], the flow rate of liquid through a tube is directly proportional to the fourth power of the radius of the tube and inversely proportional to the length of the tube and viscosity of the fluid [11].” An accurate determination of shunt volume is not feasible using echocardiography, and while, magnetic resonance imaging shows promise in measuring the volume of shunting across the ductus, its applicability in the clinical setting is not practical at present [12]. As a result, surrogate markers of pulmonary overcirculation and systemic hypoperfusion are used to estimate shunt volume and determine the degree of haemodynamic significance [11]. The overreliance of PDA diameter alone to determine PDA significance (and the sole criterion for entry into PDA treatment trials), while ignoring the other important factors including the flow pattern across the PDA, surrogate markers of pulmonary over circulation and systemic hypoperfusion, the gestation of the infant, and myocardial performance results in poor risk estimation and selection of the wrong patient population. On the one hand, infants in whom PDA treatment might be particularly beneficial may not be recruited into those trials, while on the other hand, infants in whom PDA treatment may not be beneficial (such as those destined to spontaneously close their PDA or those with bidirectional shunting indicating high PVR) may be included thereby mitigating the potential benefit of treatment. 

Currently, there is no consensus on what constitutes a haemodynamically significant PDA that is likely to be associated with adverse outcomes. Trials conducted to date utilise a heterogeneous and diverse (often not evidence based) set of clinical and echocardiographic criteria to define haemodynamic significance with considerable variability in the specific criteria and cut-off values utilised [8]. It is our opinion that, in order to characterize hemodynamic significance, a comprehensive appraisal of the cardiovascular status using echocardiography, coupled with the integration of important clinical factors, may result in an optimal risk prediction ultimately resulting in an improvement in selecting the right patient for the trials. Those elements have been described at length elsewhere but include the following components: (1) PDA shunt volume assessment and its impact on the systemic and pulmonary circulations [9]; (2) myocardial function evaluation, especially in considering how the heart handles the increased preload in the setting of potential myocardial ischemia secondary to impaired coronary artery perfusion [13]; (3) antenatal and perinatal characteristics that can act as effect modifiers to either mitigate or exacerbate potential detrimental consequences of a shunt [14].

The timing of such assessment needs consideration also. Determining PDA significance too early (within the first 24 h) may not be helpful as a certain degree of homogeneity exists between infants in relation to shunt flow due to the relatively high PVR, and as a result, distinguishing infants destined to have PDA-related complications from low risk infants likely to spontaneously close their PDA may prove challenging. Conversely, performing this assessment too late (after the first three days of age) may result a delay in identifying ideal candidates for PDA closure. 

Our group have recently devised a PDA risk score from prospective observation of a cohort of infants below 29 weeks gestation in a setting, where neither prophylaxis nor early treatment were used. A comprehensive echocardiogram performed between 36 and 48 h used markers of pulmonary over-circulation, left ventricular (LV) diastolic function, and clinical characteristics to devise a PDA severity score (PDAsc) to predict chronic lung disease or death before discharge (CLD/Death). The PDAsc had a range from 0 (low risk) to 13 (high risk). Infants who developed CLD/Death had a higher score than those who did not [7.3 (1.8) vs. 3.8 (2.0), *p* < 0.001]. A cut off PDAsc of 5.0 had an area under the curve (AUC) of 0.92 (95% CI 0.86–0.97, *p* < 0.001) for the ability to predict CLD/Death. The PDAsc cut off of 5.0 had positive and negative predictive values of 92% and 82% [10]. Incidentally, PDA diameter alone had a very poor ability (not much better than chance) to predict CLD/Death associated with a PDA. More recently, this score was tested in a pilot RCT to assess the feasibility of recruiting infants based on this risk scoring system [15]. There was a high rate of participation from the eligible infant pool with an 88% acceptance rate. The rate of adverse outcomes in infants with a low risk score (not included in the RCT, but followed up until discharge) was low providing some evidence that the PDAsc can accurately identify infants in whom the evolution of CLD/Death is unlikely. This may help in optimising the identification of high risk infants who are most likely to benefit from PDA treatment and maximise their inclusion into future PDA trials.

### 2.2. Creating True Intervention and Control Arms

One of the biggest flaws of PDA trials to date is equating “PDA treatment” with shunt elimination. The efficacy of medical PDA treatment is very variable and does not always result in achieving PDA closure and the elimination of the PDA shunt. Therefore, infants in the intervention arm often have a high rate of continued ductal patency that is often hard to quantify due to the lack of robust longitudinal echocardiography measurements in the trials. Conversely, the placebo arm is often contaminated by both, a high rate of spontaneous PDA closure and a high rate of open label PDA treatment which is often administered soon after randomisation [16]. Those characteristics often result in the two groups (intervention versus controls) being quite similar in terms of exposure to PDA shunting and medication administration. As a result comparing shunt elimination to chronic exposure of left to right shunting has never been truly been tested. 

It is important to emphasise that extrapolation of the results of these trials to imply a non-causal relationship between the PDA and physiologically attributable morbidity is incorrect. Future studies of PDA treatment should consider several important attributes that determine treatment efficacy. A “one size fits all” approach to drug selection and dosage does not result in a high rate of successful PDA closure and shunt elimination. This was recently demonstrated in our pilot RCT mentioned above where there was a very high rate of treatment failure in the intervention arm (>50%) when using the standard dosing regimen of Ibuprofen (10 mg/kg followed by 2 doses of 5 mg/kg 24 h apart). This was a particular concern in infants with the smallest gestation (<26 weeks) who are most likely to benefit from PDA shunt elimination. Those findings emphasise the important point that medication administration does not necessarily directly equate to shunt elimination in all cases. Future trials should aim to modify dosing regimens, modes of administration, and perhaps the choice of medical therapy in an attempt to achieve optimal PDA closure rates in the intervention arms where the focus should be on comparing early shunt elimination (rather that ductal treatment per se) to prolonged exposure to the PDA shunt. A further complicating factor worthy of further study is the assessment of infants in whom a partial response to PDA closure is achieved. Whether this modulation of ductal diameter and the resultant mitigation of shunt effect results in improvements of outcomes remains unknown at present. Further work is required to determine the optimal mode of administration, the dosage requirements, and the choice of drug needed to achieve a high PDA closure rate. In addition, study design should incorporate strict rules on banning open label treatment in the placebo arm to facilitate prolonged ductal exposure and the creation of two distinct groups in terms of ductal physiology, only then can we truly assess the impact of modifying shunt exposure on important neonatal outcomes. 

### 2.3. Choosing the Right Outcomes

There is an over-emphasis on two-year neurodevelopmental assessments as an important outcome measurement in neonatal PDA trials. This approach can potentially mask beneficial effects of early shunt elimination when the focus is shifted from tangible short term benefits. There are several potential drawbacks to focusing on developmental impairments at two years of age: The robustness of the two year assessment is questionable due to issues with quality control, diagnostic accuracy and the interpretation of the findings in a clinical setting. In addition, the benefit (or harm) signal measured two years after an intervention is diluted by a myriad of other arguably more important factors occurring after discharge from the hospital. As our day-to-day clinical interventions are based on achieving shorter term benefits, the primary outcomes in clinical trials should reflect that fact. We should not ignore early neonatal benefits if longer term outcomes are unmodified as a combination of potentially beneficial interventions occurring in the short term will eventually lead to an improved quality of life in the longer term [17].

Future trials of PDA treatment should adopt a set of core outcomes which are clearly defined and harmonised across different trials to facilitate better homogeneity in reporting. The timing of assessments of those outcomes should also be standardised and optimised to strengthen the temporal relationship between the intervention and the outcome measure. A recent core outcome set in neonatology was proposed for reporting in all trials including infants receiving care on the neonatal intensive care unit. Those outcomes were derived with the involvement of important stakeholders including parents and adults born prematurely and include: survival, sepsis, necrotizing enterocolitis, brain injury on imaging, retinopathy of prematurity, general gross motor ability, general cognitive ability, quality of life adverse events, visual impairment or blindness and chronic lung disease (CLD) [18]. The dichotomy of the CLD diagnosis can pose further challenges in determining benefit following PDA shunt elimination. Like a PDA, respiratory morbidity is likely to lie on a physiological continuum and more graded assessment may prove more beneficial when implemented in a trial setting. In addition, characterising respiratory morbidity over the first year of age may also be a worthy outcome worth pursuing in extremely premature infants. Recently, a Post Prematurity Respiratory Disease (PRD) was described in premature infants which encompasses respiratory related hospitalisations, home support (oxygen, tracheostomy, and home ventilation), medications and persistent coughing/wheezing [19]. Incorporating PRD in future PDA trials would be a welcome move due its relevance to the quality of life of premature infants and their families. Sample size planning should take the short term outcome into consideration while the longer term outcomes should be reserved as a safety assessment measure in future trials. 

## 3. Conclusions

It becoming increasingly evident that trials, to date, involving PDA treatment have failed to demonstrate an improvement in outcomes following PDA treatment. However, due to the shortcomings explained above, we cannot extrapolate that certain PDAs at least are inconsequential and require no intervention. In order to definitively address the PDA conundrum, future trials should focus on the following:

(1) Ensuring that infants at the highest risk of PDA related morbidities with a low chance of spontaneous closure and are most likely to benefit from treatment are included in the trials. This requires a robust and comprehensive echocardiography assessment of PDA physiology incorporating aspects of myocardial performance with important clinical features integrated into the risk assessment. Reliance on single markers for inclusion to trials (such as PDA diameter alone) should no longer be implemented.
(2) The timing of enrolment and intervention should balance avoiding intervention that may be too early, which could result in overtreatment and potentially dilute beneficial effects of shunt elimination in high risk infants, avoiding late treatment where prolonged shunt exposure may cause irreversible damage.
(3) Treatment regiments in the intervention arm should focus on optimising PDA closure and echocardiography confirmed shunt elimination, while banning open label treatment in the placebo arm is vital to ensure ductal patency persists in this group of infants. This approach will facilitate a true comparison between early shunt elimination versus chronic exposure to left to right shunting.
(4) Selecting outcome measures relevant to infants and parents with the focus on reducing important short term morbidities while reserving longer term neurodevelopmental impairment as a safety measure in those trials.

Until those conditions are met in future PDA trials, we are destined to suffer the same initial fate as Phil Connors in Groundhog Day.

## Data Availability

Data sharing not applicable.
